# A Pilot Study Combining a GC-Sensor Device with a Statistical Model for the Identification of Bladder Cancer from Urine Headspace

**DOI:** 10.1371/journal.pone.0069602

**Published:** 2013-07-08

**Authors:** Tanzeela Khalid, Paul White, Ben De Lacy Costello, Raj Persad, Richard Ewen, Emmanuel Johnson, Chris S. Probert, Norman Ratcliffe

**Affiliations:** 1 Institute of Translational Medicine, University of Liverpool, Liverpool, United Kingdom; 2 Institute of Biosensor Technology, University of the West of England, Frenchay, Bristol, United Kingdom; 3 Bristol Urological Institute, North Bristol NHS Trust, Bristol, United Kingdom; University of California, Merced, United States of America

## Abstract

There is a need to reduce the number of cystoscopies on patients with haematuria. Presently there are no reliable biomarkers to screen for bladder cancer. In this paper, we evaluate a new simple in–house fabricated, GC-sensor device in the diagnosis of bladder cancer based on volatiles. Sensor outputs from 98 urine samples were used to build and test diagnostic models. Samples were taken from 24 patients with transitional (urothelial) cell carcinoma (age 27-91 years, median 71 years) and 74 controls presenting with urological symptoms, but without a urological malignancy (age 29-86 years, median 64 years); results were analysed using two statistical approaches to assess the robustness of the methodology. A two-group linear discriminant analysis method using a total of 9 time points (which equates to 9 biomarkers) correctly assigned 24/24 (100%) of cancer cases and 70/74 (94.6%) controls. Under leave-one-out cross-validation 23/24 (95.8%) of cancer cases were correctly predicted with 69/74 (93.2%) of controls. For partial least squares discriminant analysis, the correct leave-one-out cross-validation prediction values were 95.8% (cancer cases) and 94.6% (controls). These data are an improvement on those reported by other groups studying headspace gases and also superior to current clinical techniques. This new device shows potential for the diagnosis of bladder cancer, but the data must be reproduced in a larger study.

## Introduction

Over 10,000 patients are diagnosed with bladder cancer in the UK annually and half will die of the disease [1]. Most patients (80%) present with painless haematuria which must be investigated [2]. Of those with visible haematuria 19% will have bladder cancer while for non-visible haematuria the rate is just 5% [3]. Haematuria, although important, has a low pre-test probability of diagnosing bladder cancer for which there are no screening tests. Consequently, cystoscopy remains the gold standard for detecting the disease in patients with haematuria [4]. Cystoscopy is a frequently performed procedure and the cost to the NHS, of investigating people found not to have bladder cancer, is £33.5M per year [3], based on a tariff currently of around £400. Cystoscopy is a costly, invasive and undignified procedure. There is a need for an inexpensive, less invasive alternative.

Bladder cancer cases presenting at an early stage can be treated by transurethral resection without the need for major extirpative surgery. Furthermore this early stage disease has an excellent 5-year survival rate (>94%) compared to <50% 5-year survival for more advanced disease, so a non-invasive screening test that identifies cancer at an early stage from urine headspace has the potential to revolutionise patient care. Despite resection, the majority (50-70%) will recur and 10-30% progress to advanced disease [5], consequently frequent cystoscopy checks are necessary which account for 71% of the costs of treating bladder cancer [6]. These factors mean that bladder cancer is the most expensive cancer per patient to manage [7]. Various mass screening options have been considered, including the haematuria dipstick, NMP22, or UroVysion [8]. However, these biomarkers, as well as urine cytology, lack sensitivity [9]. Currently, no biomarkers can be recommended for clinical practice because of their poor sensitivity and specificity, although there is some evidence that biomarkers may be of use in monitoring recurrence or screening high risk individuals [10]. New selective biomarker(s) for bladder cancer would reduce diagnostic uncertainty and surveillance-related morbidity. It could reduce costs and improve patients’ quality of life by avoiding unnecessary invasive diagnostic tests. It could also improve prognosis for patients through earlier detection of potentially lethal disease whilst decreasing the economic burden of haematuria clinics [11]. There is some evidence to support the hypothesis that bladder cancer is associated with the presence of specific volatile organic compounds (VOCs) in the gas emitted from urine samples. In 1999 Spanel et al. reported that formaldehyde was more abundant in the urine of men with bladder cancer than from those with prostate cancer and healthy controls [12]. A study in which dogs were trained to respond to the urine samples from patients with bladder cancer gave a mean success rate of 41% compared to the 14% expected by chance [13]. More recently the same group reported that the best performing dog could identify 73% of cancers [14]. Meanwhile, VOC analysis using e-nose technology was found to have 70% sensitivity and specificity for the diagnosis of bladder cancer from healthy controls, dropping to 60% and 67% respectively when testing with urine controls from patients with non-malignant urological diseases [15]. In this report, we describe data from a pilot study which shows the potential for a novel, in-house fabricated VOC sensor device for accurately diagnosing bladder cancer.

## Methods

### Patient Recruitment

A total of 98 male patients, aged 27-91 years, presenting to urology clinics in Bristol were recruited after obtaining informed consent. All had undergone a clinical assessment for their symptoms. Demographics for the patient groups studied are given in Table 1; there were 3 controls per cancer patient. Each patient provided a urine sample on the morning prior to their examination or cystoscopy. Samples were classified when the clinical assessment was completed. When bladder cancer (n=24) was found, grade and stage of the cancer were recorded (Table 2); the majority were well-differentiated or intermediate grade (G_1_, G_2_) and at an early stage (T_a_) confined to the bladder lining. There were 74 controls, patients who were undergoing investigation for symptoms of bladder outflow obstruction and or haematuria; their symptoms and relevant co-morbidities included: 31 had raised PSA without malignancy, 24 had hypertension, 19 had visible haematuria, 15 had bladder outflow symptoms, 7 diabetes, 4 non-visible haematuria, 2 had renal tract stones and 1 had undergone renal transplant surgery.

**Table 1:** 

**Diagnosis**	**No. of patients**	**Age range in years (median)**	**Smoking status**
Bladder cancer	24	27 – 91 (mean 71)	7 smokers; 17 non-smokers (9 ex-smokers)
Control (non-cancer)	74	29 – 86 (mean 64)	15 smokers; 59 non-smokers (17 ex-smokers)

**Table 2:** 

**Tumour**	**Tumour stage (TNM)**		
**Grade**	**T_a_N_0_M_x_**	**T_1_N_0_M_x_**	**T_2_N_0_M_x_**
G_1_	6	Nil	Nil
G_2_	10	1	Nil
G_3_	1	5	1

The tumours were graded according to the WHO 1973 guidelines. G_1_: well differentiated (look similar to the normal cells found in the bladder - slow growing cancer) G_2_: intermediate level of differentiation (mixture of G_1_ & G_3_) G_3_: poorly differentiated (looks very different from normal bladder cells – most aggressive). Staging of the tumour is given according to the TNM (Tumour, Node, Metastasis) criteria. T_a_: the cancer is just in the innermost layer of the bladder lining. T_1_: the cancer has started to grow into the connective tissue beneath the bladder lining. T_2_: the cancer has grown through the connective tissue into the muscle. All patients are also graded N_0_ (No regional lymph-node involvement) M_x_ (metastasis status was not assessed).

Ethical approval for the study was obtained from the Wiltshire Research Ethics Committee; each participant reviewed an information sheet and gave written consent.

### Sample storage

*Aliquots of fresh* urine (0.75 ml) were transferred to septum-topped, glass headspace vials (Sigma Aldrich, Dorset, UK) and were frozen at -20^o^C until analysis. In a previous study we found that freezing had no detectable effect on the volatile composition of the samples [16].

### Headspace Analysis

Each urine sample was defrosted by immersing the vial in a water bath at 60^o^C for 30 seconds. Thereafter, each sample was treated with an equal volume (0.75 ml) of sodium hydroxide (1M; Fisher Scientific, Leicestershire, UK) and the mixture equilibrated at 60^o^C in a water bath for 50 minutes prior to extraction of 2cm^3^ of headspace air. Headspace air was extracted using an airtight Hamilton gas syringe (10ml, Fisher Scientific, Leicestershire, UK) and was then immediately injected into the inlet of a gas chromatograph coupled to a sensor detection system.

The VOCs from urine samples were analyzed using an in-house fabricated sensor system. The device comprised of a conventional gas chromatography oven (Clarus 500, PerkinElmer) fitted with a commercially available capillary column (30 metre long SPB^TM^-1 SULFUR capillary column with an inner diameter of 0.32 mm and a film thickness of 4 μm (Sigma Aldrich, Dorset, UK)) which was interfaced to a heated (450^o^C) metal oxide sensor used as the detector. The metal oxide sensor consisted of a tin oxide, zinc oxide mix (50: 50 wt%). The sensor preparation and characterisation has been reported elsewhere [17]. The GC injector was heated to a preset temperature of 150^o^C and was fitted with a 1mm quartz liner. Compressed air (BOC, Guilford, UK) was used as the carrier gas at a pressure of 35 psi and was passed through an air purifier (300ml Supelcarb^TM^ Hydrocarbon trap, Sigma-Aldrich, Dorset, UK). The GC temperature programme used was as follows: initial GC oven temperature held at 30^o^C for 6 minutes, then ramped at a rate of 5^o^C/min to 100^o^C with a 22 minute hold, giving a total run time of 42 minutes.

The GC sensor system was calibrated daily using a certified gas standard (1% tolerance) of 50 ppm ethanol in blended air (Air Products Plc, Speciality Gases, Crewe CW1 6AP).

After headspace gas (2cm^3^) was injected into the device the volatile compounds were separated according to their chemical properties and eluted from the column at specific times. Volatiles eluted from the column and impinged on the sensor. The volatile compounds adsorbed and reacted on the heated metal oxide surface of the sensor concomitantly producing a reversible change in the electrical resistance. The change in electrical resistance versus time profile for the sensor produced a sample specific chromatogram. These resistance time chromatograms were then interpreted statistically based on their group membership.

### Statistical analysis

Resistance was sampled at intervals of 0.5 seconds for 42 minutes (t = 1, 2,…. , 5040). The utility of highly specific features of the profiles, low order frequencies in the profiles, and broader profile features of the data in developing a good classifier was investigated. As there is no gold standard approach to data of this kind, we investigated two independent methods of statistical analysis.

Methods 1 and 2 were based on standardizing each resistance profile so that each had a mean value of 0 and a standard deviation of 1 [15]. This transformation ensures each donor profile is on the same scale permitting a fair comparison between disease groups, invariant of scale and location effects, whilst preserving the relative minima and maxima within each profile. Statistical validation of models was undertaken using leave-one-out cross-validation which provides an indication of model robustness and predictive performance on fresh unseen samples.

#### Method 1

This was based on a between-groups comparison of the standardized resistance profiles using two-group linear discriminant analysis implementing the forward stepwise selection algorithm to ensure a) the number of predictors in the model does not exceed the smallest group size and b) the inclusion of a predictor variable does not result in multi-collinearity problems. The within-sample robustness of the predictive properties of the derived model was assessed using leave-one-out cross validation. This modelling strategy was designed to investigate whether a simple parsimonious description of between-groups separation existed based upon highly specific differences in profiles between groups.

#### Method 2

Partial least squares discriminant analysis (PLS-DA) was used to build a two group classifier for the discrimination of bladder cancer cases from controls. PLS-DA is an established supervised classification technique used in sensormetric analyses and is an information extraction and data reduction technique designed to maximally separate two or more groups and is particularly suited to high dimensional and multi-collinear data. This approach is considered to be better than alternative approaches using principal components analysis over a wide range of classification problems [18]. In this context, PLS-DA was considered from a proof-of-concept aspect i.e. can a small number of latent factors extracted from the entire data set discriminate between cases and controls. We adopted an extraction procedure using up to ten latent factors and used a leave-one-out classification to assess within sample classification accuracy.

## Results

Sensor outputs were compared in the two groups; patients with bladder cancer (24) and patients without cancer (74). Two independent statistical approaches were taken to determine whether there were differences in the sensor output. For each method, patients with cancer were compared with those without; the sample size was too small to be able to compare patterns with grade or stage (Table 1).

**Figure 1:**
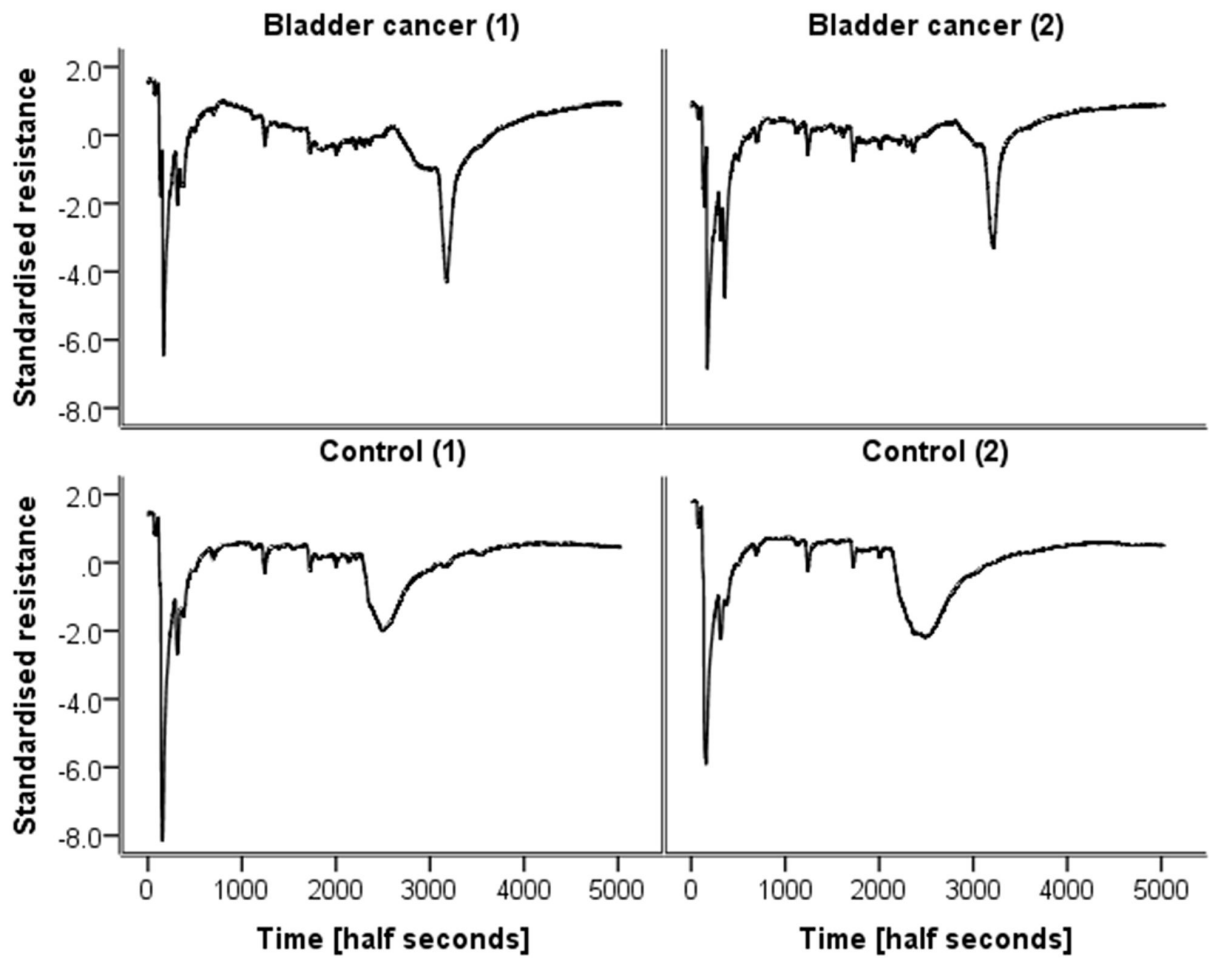
Examples of chromatograms (standardised resistance vs. time) of the urine of two patients with bladder cancer and two controls.

**Figure 2:**
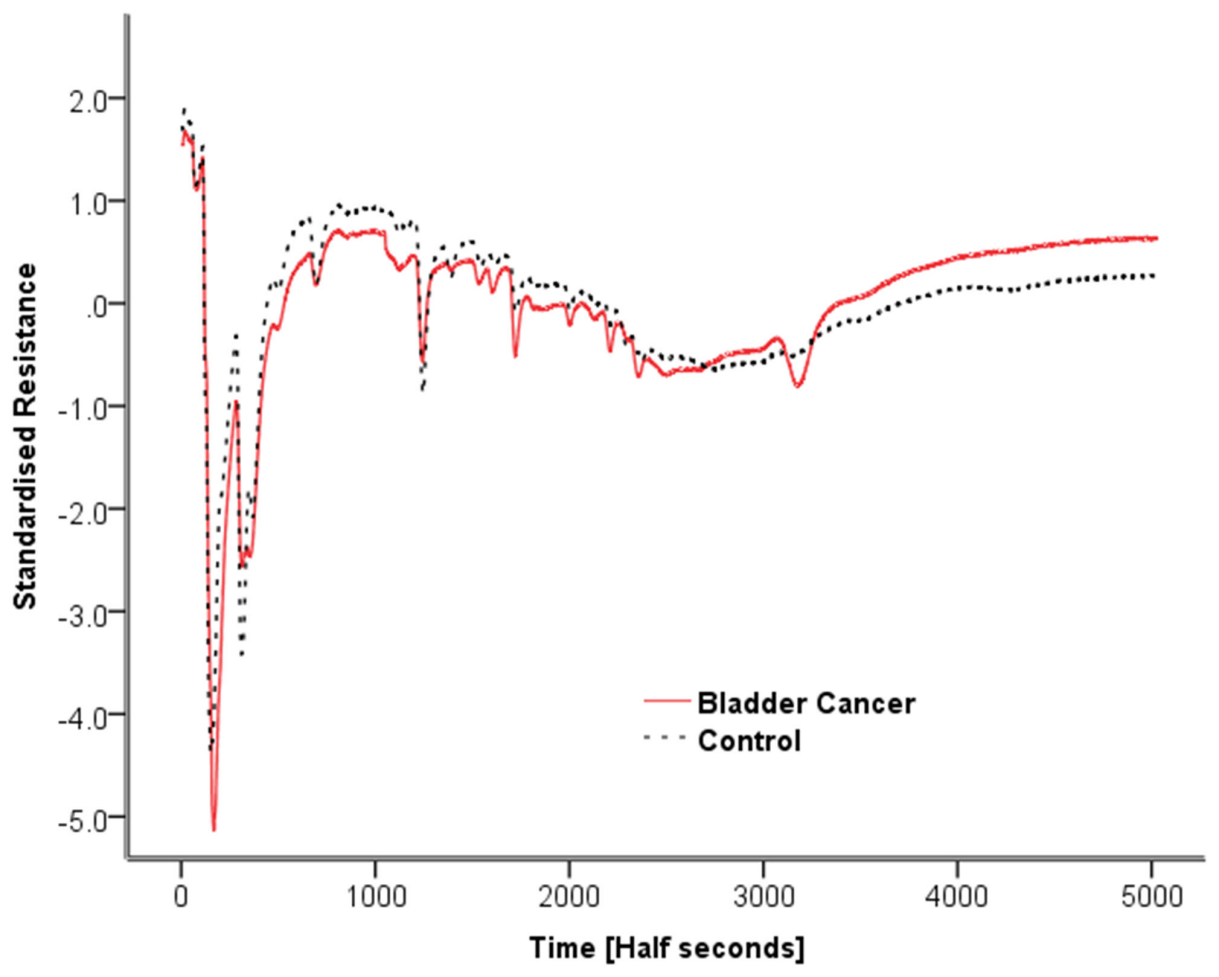
The averaged chromatograms for the bladder cancer group and the control group shown on the same standardised resistance time plot.

Figure 1 shows typical chromatograms of standardised resistance versus time for two controls and two bladder cancer patients. The chromatograms of the two bladder cancer patient samples are broadly similar as are those of the two control samples. However, it is apparent that the control samples differ from the bladder cancer samples. The most obvious difference is a peak at circa 3200 (half seconds) which is present in both bladder cancer samples but absent from both controls. Analysis of the chromatograms showed that 20/24 bladder cancer samples had a measurable peak in this region. In the control samples, peaks in this region of the spectra were predominantly absent. To highlight this point, Figure 2 shows overlaid averaged chromatograms for each group, clearly showing the feature at 3200 half seconds in the bladder cancer group, which is absent from the control group.

**Figure 3:**
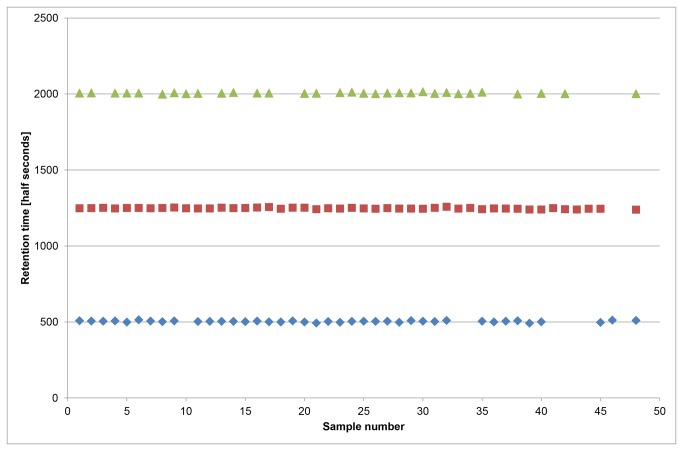
The retention time stability of three common peaks from 24 selected control samples and the 24 bladder cancer samples, in date order. There is a minimal day to day fluctuation *circa* ± 1%.

Figure 3 shows the retention time stability of the system for three common peaks which span the retention time range where the majority of the detected peaks elute. The data shown are for 24 selected control samples (which span the full 7 month duration of the study) and the 24 bladder cancer samples. There is no noticeable trend in terms of drift over time (samples 1-24 controls and 25-48 bladder cancer are displayed in run date order). There is a day to day fluctuation in the measured retention times of *circa* ±1%. In addition an ethanol standard was run daily in order to assess the retention time and sensitivity of the system. The retention time of ethanol over the course of the study had a mean of 329.2 half seconds (S.D. 3.97), in line with the results from the in sample peaks. The average width of the peaks is circa 100 half seconds and can be up to 200 half seconds at higher retention times. Therefore, variability of approximately ±1% was found to have a negligible effect on the predictive accuracy of the statistical models (see section Statistical Analysis Method 1 below).

The average sensitivity to ethanol over a two month period was 98.5% (S.D. 10.6) (R_0_-R_g_/R_0_ ×100, where R_0_ is the baseline resistance and R_g_ is the resistance when exposed to ethanol). Although the sensitivity of the sensor was maintained at a reasonably constant level the baseline resistance was subject to drift. To counteract this effect and to aid visual and statistical comparison of the results the resistance scale was standardised using the methodology described above.

### Statistical Analysis Method 1

A total of 9 time points were selected (t (sampling time points) = 117, 153, 174, 201, 359, 1073, 1162, 1362, 3204). Within samples 24/24 (100%) of bladder cancer cases and 70/74 (94.6%) controls were correctly assigned. This means the model detected the cancer in all patients, irrespective of the grade or stage. Under leave-one-out cross validation these values fell to 23/24 (95.8%) and 69/74 (93.2%) correct classification, respectively (Table 3). Other discriminant models give similar predictive accuracy (e.g. the sampling time points t = 117, 201, 231, 362, 1090, 1360, 3297, 3334, 3417 gives cross-validated accuracy of 95.8% for bladder cancer cases, and 94.6% for controls). This shows that the model is not sensitive to specific “retention times”. It also shows that the time points selected do not necessarily represent separate peaks, but are simply time points where the two groups of spectra differ maximally. In this second model 3 time points are selected which span the broad peak present in the bladder cancer group.

**Table 3:** Classification results using two-group linear discriminant analysis.

		**Predicted group membership**		
		**Bladder cancer (%)**	**Control (%)**	**Total**
**Original model**	**Bladder cancer**	24 (100)	0 (0)	24
	**Control**	4 (5.4)	70 (94.6)	74
**Cross-validation (leave-one-out)**	**Bladder cancer**	23 (95.8)	1 (4.2)	24
	**Control**	5 (6.8)	69 (93.2)	74

### Statistical Analysis Method 2

The results of PLS-DA in terms of percentage correctly classified within sample and in using leave-one-out classification are given in Table 4 according to number of latent factors extracted in the modelling process. The use of 7 or more factors identified 23/24 of the patients with cancer: again suggesting the diagnosis could be made in the vast majority of patients, irrespective of the grade or stage of the cancer.

**Table 4:** Discrimination of bladder cancer cases from controls using PLS-DA.

**Number of latent factors**	**Predicted correct**		**Predicted correct**	
	**Within sample**		**Leave-one-out**	
	**Bladder (%)**	**Control (%)**	**Bladder (%)**	**Control (%)**
1	19 (79.2)	46 (62.2)	19 (79.2)	46 (62.2)
2	18 (75)	60 (81.1)	18 (75)	59 (79.7)
3	21 (87.5)	63 (85.5)	20 (83.3)	64 (86.5)
4	21 (87.5)	64 (86.5)	21 (87.5)	64 (86.5)
5	23 (95.8)	62 (83.8)	23 (95.8)	61 (82.4)
6	22 (91.7)	68 (91.9)	21 (87.5)	67 (90.5)
7	23 (95.8)	70 (94.6)	23 (95.8)	67 (90.5)
8	23 (95.8)	70 (94.6)	23 (95.8)	69 (93.2)
9	23 (95.8)	71 (95.9)	23 (95.8)	71 (95.9)
10	23 (95.8)	71 (95.9)	23 (95.8)	70 (94.6)

Abbreviation: PLS-DA, partial least squares discriminant analysis

## Discussion

A new technique is described for the identification of patients with bladder cancer based on the profile of volatile organic compounds emitted from their urine, using a GC combined with a sensor system. The findings build on previous work using dogs [14] and an electronic nose [15] which provide proof of concept data for our findings.

Our findings are significantly better than those described previously using the volatile analysis approach. The improvement is probably the result of two factors: 1) use of the chromatography separation step and 2) the adjustment of pH using base. Base would have had several effects, the high ionic strength would have driven VOCs into the headspace and it would also have suppressed acids, increasing the concentration of basic compounds in the headspace. Previous work by our group has found that base treatment of urine samples (compared to acid treatment or untreated samples), enabled the identification of biomarkers that allowed differentiation between prostate cancer and controls [19].

Sensor outputs were analyzed using two different, independent data analysis techniques each of which showed significant discrimination of samples from patients with and without bladder cancer. Control samples were collected from patients who had attended clinic with urological problems that were later found to be non-malignant. The choice of controls is important to assess the clinical usefulness of such a test. Patients without cancer may present similar symptoms to those with cancer and it is differentiating these that are the clinical challenge. Others [15] have reported that patients with bladder cancer could be differentiated from healthy controls with greater certainty than controls from urology clinics. However, we have specifically chosen controls recruited from the same urology clinic: many of them had haematuria making them ideal controls for this study.

Two different statistical approaches were used, linear discriminant analysis and partial least squares discriminant analysis, however, both techniques used slices of the time profile (bins). The results were similar; using 6 or more components in the PLS-DA correctly identified over 90% of cases and controls, while the simple linear DA correctly classified over 93% of cases and controls.

A review of biomarkers for the detection and surveillance of bladder cancers [20] gave sensitivity and specificity ranges, respectively, for each marker as follows: bladder tumour antigen (BTA), BTA Stat (Polymedco), 52.5%-78.0% and 69.0%–87.1%; BTA Trak (Polymedco), 51%-100% and 73%–92.5%; cytology, 12.1%-84.6% and 78.0%–100%; hematuria dipstick, 47.0%-92.6% and 51.0%–84.0%; nuclear matrix protein (NMP), NMP22 Bladder Cancer Test (Matritech), 34.6%-100% and 60.0%–95.0%; NMP22 BladderChek (Matritech), 49.5%-65.0% and 40.0%–89.8%; ImmunoCyt/uCyt+ (DiagnoCure), 63.3%-84.9% and 62.0%–78.1%; ImmunoCyt/uCyt+ and cytology, 81.0%-89.3% and 61.0%–77.7%; and UroVysion (Abbott Molecular)/florescence in situ hybridization, 68.6%-100% and 65.0%–96.0%. As can be observed there is a wide range of results. The review concluded that no currently available bladder cancer urinary marker is sensitive enough to eliminate the need for cystoscopy. A recently published algorithm, combining tests, suggested this may increase sensitivity and specificity to 91% and 80% respectively, but the paper concluded that it remained a challenge to accurately detect all bladder cancers [9]. Our technique for bladder tumour diagnosis gives results that are comparable and, in some cases, better than currently available urinary biomarkers for bladder cancer. The GC-sensor approach is low cost and can be used by semi-skilled personnel. The sensor is relatively inexpensive to fabricate and its lifetime is likely to be far in excess of 100 analyses. Furthermore the components are capable of further miniaturization to produce a point of care device. For example miniaturised GC systems already exist that make use of microelectromechanical system (MEMS) components [21]. The combination of the GC separation element and sensor element on a MEMS platform is also an active area of research [22,23]. The current device uses a minimal temperature ramp but it may be possible to perform the separation isothermally at ambient temperatures. It also uses air as the carrier gas introducing the possibility of using a compressor with appropriate filters instead of gas cylinders.

The reason for the presence of volatile biomarkers is probably a change in metabolism by malignant cells. There is evidence for oxidative stress and induction of cytochrome P450 by lung, breast, and prostate cancers [24-27]; this is associated with a change in exhaled VOCs from patients with lung and breast cancer [24,25]. Studies of bladder cancer have also found altered enzymatic activity [28] as well as abnormalities of the citric acid cycle [29], resulting in increased conversion of pyruvic acid to lactic acid. How these two examples result in the production of VOCs is not clear, but they serve to illustrate that there are abnormalities of particular metabolic pathways with specific changes in metabolites as a result. In the case of the bladder, the proximity of malignant cells to urine is likely to result in abnormal VOC patterns in urine of patients with bladder cancer. In summary the use of the GC-sensor system for detection of VOC profiles speeds up the process of data analysis compared to the much more laborious and expensive systems such as GCMS which also require highly experienced researchers.

The identity of the VOCs that contribute to the biomarker profile has yet to be determined. Their characterisation may be defined in future work using gas chromatography mass spectrometry techniques and this will provide further insight into bladder cancer induced biochemical changes.

The study is a small scale prospective investigation based around the intuitively appealing proposition of the existence of some systematic compositional differences in urine samples between those patients with, and those patients without, bladder cancer. In this research the treated urine samples are quantitatively characterised using a reliable in-house fabricated sensor coupled with gas chromatographic separation.

The research is particularly challenging and retains good ecological validity by not using healthy controls. Results are extremely encouraging. However, the relatively small sample size is based on patients from a single centre and population coverage and external validity cannot be assured; consequently some caution must be exercised with regard to generalising beyond the sample. It is also recognised that the robustness of statistical models based on small sample sizes could be prone to the presence of any chance idiosyncratic sample features, and the relatively small sample sizes do not readily permit stratification on potentially important patient features (e.g. tumour size). Equally the small sample size does not allow a proper statistical assessment of the effects of other confounding factors such as smoking status, drug treatments, ethnicity etc. In addition an all male cohort was selected as the basis of the study, this removes a confounding factor but is a limitation of the current study despite a much higher incidence of this cancer in the male vs. female population (circa 2.5:1). Another limitation of the current study concerns the use of frozen samples, although we have collected robust data from GC-MS studies that freezing does not have a marked effect on the volatile profile [16]. In terms of the device, the long term stability of the sensor and retention time drift are potential limitations. The use of certified gas standards in this study ensured that the sensitivity of the sensor was regularly monitored and maintained at a comparable level. Retention time was subject to fluctuations of ±1%, this was assessed by measuring common peaks in the sample and the retention time of the ethanol standard over time. As the peaks in the chromatogram are relatively broad (when compared to the possible error) and the statistical model is not dependent on identifying single peaks with specific retention times, we are confident that this error does not unduly affect the current analysis. In longer term studies a standard mixture of compounds spanning the retention time range could be substituted for ethanol, allowing for correction of any drift.

Leave-one-out cross validation coupled with the triangulation of results from markedly different statistical analysis techniques provide good *prima facie* support for the broad claim of differences between groups within the collected samples.

## Conclusion

The novel combination of a GC and a unique metal oxide sensor based device has provided data to underpin a new instrument for the diagnosis of bladder cancer. The statistical model could be easily used to write an algorithm that will display the diagnosis. Both of the models tested were 96% accurate in diagnosing bladder cancer. These data must now be reproduced in larger studies.
